# Using an Automated Data-driven, EHR-Embedded Program for Mailing FIT kits: Lessons from the STOP CRC Pilot Study

**DOI:** 10.4172/2329-9126.1000141

**Published:** 2014-01-05

**Authors:** Gloria D. Coronado, Tim Burdick, Amanda Petrik, Tanya Kapka, Sally Retecki, Beverly Green

**Affiliations:** 1 Kaiser Permanente Center for Health Research, USA; 2 OCHIN, USA; 3 Virginia Garcia Memorial Health Center, USA; 4 Group Health Research Institute, USA

**Keywords:** Electronic health record, Colorectal cancer screening, Reporting workbench, Patient registry, Clinical decision support, Fecal immunochemical (FIT) kit

## Abstract

**Background:**

The Strategies and Opportunities to Stop Colorectal Cancer (STOP CRC) study is collaboration among two research institutions and health-systems partners. The main study, scheduled to begin in 2014, will assess effectiveness of an intervention program using electronic health record (EHR) clinical decision support (CDS) tools to improve rates of colorectal-cancer screening in federally qualified health centers (FQHCs). Very few studies, and no large studies, aimed at raising CRC screening rates have utilized an EHR-embedded system.

**Study design:**

We piloted the use of an EHR-embedded real-time patient registry reporting tool in a pilot study undertaken prior to beginning our main CRC screening study. The pilot study goal was to assess feasibility and effectiveness of two clinic-based approaches to raising rates of colorectal cancer screening among selected patients aged 50–74 who were not up-to-date with colorectal-cancer screening guidelines. We used work sessions and qualitative interviews with clinic personnel to assess performance of the tool, as well as to identify specific elements of the tool’s functionality needing refinement.

**Results:**

Two critical elements of the EHR tool allowed us to mail FIT kits efficiently to appropriate patients: (1) having a direct interface with the laboratory that processed the FITs, thus allowing for real-time updates to the registry; and (2) being able to place lab orders from a list of selected patients. We identified the following elements that needed refining: the use of Health Maintenance (EHR function for tracking screening eligibility and due dates incorporating STOP CRC inclusion and exclusion criteria), and the development of report templates for identifying patients eligible for each step.

**Conclusion:**

We found that most elements of our EHR-embedded program worked well and that specific refinement may improve the accuracy of identifying patients at each step. Our findings can inform future efforts to build EHR-embedded CDS tools for preventive services.

## Introduction

Colorectal cancer (CRC) is the second-leading cause of cancer deaths [1]. In 2013, an estimated 142,000 adults in the U.S. will be diagnosed with CRC and 51,000 will die from the disease [2,3]. Accelerating adoption of screening could reduce CRC mortality more than 50% by 2020 [4]. Despite the clear benefits of screening, data from the National Health Interview Survey (NHIS) show that, in 2010, 41% of adults aged 50–75—nearly 35 million people—were not up-to-date with CRC screening recommendations [5]. Almost 30% of eligible adults have never had any type of CRC screening [6]. These rates are well below goals set by the American Cancer Society (75% by 2015) [1] and by Healthy People 2020 (70.5%) [7].

Klabunde and others have noted that primary-care practices play a critical role in achieving public-health targets for colorectal cancer screening; provider recommendation is strongly correlated to patient receipt of CRC screening [8,9] and previous studies show that practice-level systems to support the translation of provider recommendation into care delivery are important influences on CRC screening uptake [10,11]. Yet, the adoption of practice-level systems for CRC screening is slow, and primary-care providers often lack systematic clinical decision support (CDS) methods for identifying patients eligible for screening, as well as time and reimbursement for counseling about screening choices. There are also few systems that track receipt of CRC screening tests; ensure evaluation of abnormal results; or use CDS to improve follow-up testing at appropriate intervals [12].

Previous evaluations of clinic-based programs to improve rates of CRC screening have shown that direct mailing of guaiac fecal occult blood tests (gFOBT) or fecal immunochemical tests (FIT) consistently led to 6–24% increases in CRC screening rates regardless of clinical setting [10,13–15]. While some studies have used Electronic Health Record (EHR) alerts to increase CRC screening rates, none of the previous mailed interventions embedded their registry functions directly into the electronic health record (EHR) and into existing clinical staff workflows. This lack of integration diminishes the opportunity for sustaining the intervention programs over time and for studying these programs’ reach and effectiveness.

Strategies and Opportunities to STOP Colon Cancer in Priority Populations (STOP CRC) is an NIH-sponsored Health Systems Collaboratory Demonstration Project (UH2AT007782) that uses a cluster-randomized pragmatic design to test automated strategies to raise rates of colorectal-cancer screening in federally qualified health center (FQHC) clinics, sites where CRC screening rates historically are very low. The intervention consists of an automated data-driven, EHR-embedded program for mailing FIT kits to patients due for CRC screening. We have previously reported on effectiveness findings from the STOP CRC pilot study, conducted in partnership with Virginia Garcia Memorial Health Center (VGMHC). (Coronado; in press) In this paper, we describe details about the EHR-based population management tool we used in the pilot study, as well as how well the tool worked to serve the primary aims of the study. In addition, we describe the feedback we received from clinic staff that led to design changes in the tool for future use in the main study. Ours is the first study to report on use of an EHR-based CDS tool in an FQHC population to identify appropriate patients and to track CRC screening receipt and follow up.

## Materials and Methods

Impetus for Conducting a CRC-screening Effectiveness Study Using EHR-based Tool.

In the OCHIN population, there was no automated tool to track delivery and completion of CRC tests, and there had been no systematic push to increase screening rates through fecal testing. Moreover, when patients received fecal tests there were no systems in place to remind them to return them for processing. These factors, and our interest in launching a program that could be sustained and evaluated over time, were the drivers behind our study to use an automated EHR-based tool to raise CRC-screening rates in this FHQC population.

## Participants/patients

The first-phase pilot-test of the CRC screening program was conducted in two clinics within the larger Virginia Garcia Memorial health Center (VGMHC) organization. In 2012, VGMHC had 5,190 active patients aged 50–74, of whom 46% were Hispanic and 59% were uninsured. The pilot study recruited 213 patients aged 50–74, who received care in the past year at either of the two participating intervention clinics of VGMHC, and who were not up-to-date with recommendations for colorectal-cancer screening.

## Pilot effectiveness results

STOP CRC consists of a pilot study and a larger multi-site pragmatic study which will begin in 2014. The pilot study compared two clinic-based interventions: (1) an automated data-driven, EHR CDS tools for mailing FIT kits (with pictographic instructions [16] and return postage) to patients due for CRC screening (Auto Intervention); and (2) a higher-intensity program consisting of a mailed FIT kit plus linguistically and culturally tailored interventions delivered at the clinic level that accounts for individual clinics’ resources, capacity, and preferences (Auto Plus Intervention) to a usual-care clinic. The interventions were designed to encourage patients to complete home-based FIT testing.

**Figure 1:**
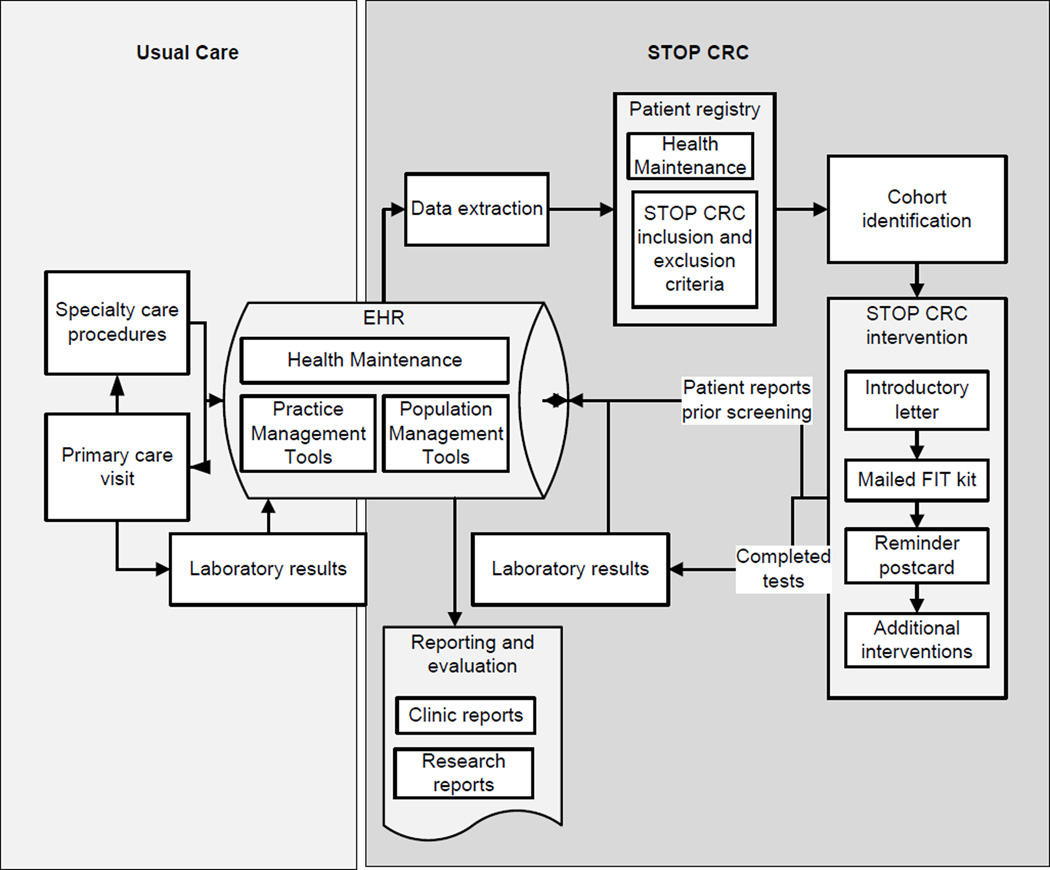
Schematic of EHR data flow for STOP CRC.

The pilot results showed that, on average, CRC fecal testing screening rates in the two intervention sites were 38% (39% in the Auto intervention and 37% in the Auto Plus intervention) at 6 months, compared with 1% in the usual care clinic. Usual care for CRC screening involves “opportunistic screening”; that is, CRC screening that is offered during a clinical encounter (Figure 1). The STOP CRC intervention, in contrast, was conducted outside of the clinical encounter and relied on mailed FIT kits to patient homes. Patients then mailed kits directly to the laboratory, where they were processed and results delivered via electonic interface into the EHR.

## STOP CRC’s embedded EHR CDS tools

We worked with staff from VGMHC (an EMR site specialist, the operations director, and a primary-care provider) to develop functional requirements for an EHR population management tool. Analysts from OCHIN then configured the Epic^©^ EHR software (version 2010; Verona, WI). The primary CDS tool was Reporting Workbench, an integrated, real-time, patient registry of patients due for CRC screening. Specifically, we defined the codes to identify an initial set of eligible patients to create the registry; then we identified data fields to filter patients and to create sub-registries of patients eligible for subsequent intervention steps.

## Design

### Ethical considerations

The study has been approved by the Institutional Review Board of the Kaiser Permanente Northwest Center for Health Research (CHR-NW). The larger multi-site pragmatic study will be implemented in 24 FQHC clinics.

### Creating an EHR-Embedded patient registry

OCHIN staff used Reporting Workbench to create a registry of patients aged 50–74 who were eligible for CRC screening. The clinic and research team determined eligibility and exclusionary criteria to identify patients to target for screening interventions. These criteria excluded those with EHR evidence of being up-to-date with CRC screening recommendations (FOBT within 1 year, flexible sigmoidoscopy within 4 years, or colonoscopy within 9 years) or of having a limited set of health conditions (e.g. prior CRC, inflammatory bowel disease, renal failure). Clinics opted to exclude patients who had an order for FOBT in the prior year that had not been completed. The registry also excluded patients with a referral to gastroenterology in the past year. OCHIN staff applied automated codes from the EHR to create an initial registry.

Once the registry had identified patients eligible for CRC screening, the intervention consisted of 3 sequential mailings: (1) an introductory letter; (2) a FIT kit; and (3) a reminder postcard. In the Auto-Plus clinic, clinic staff conducted an additional intervention activity, a live phone-call reminder that uses motivational interviewing. After modification for our study, Reporting Workbench was able to exclude patients from additional solicitations for screening for a variety of reasons (having already completed screening, reporting previous screening events that made them up-to-date with screening recommendations, declining participation, or having an invalid address).

#### Step 1: Mailing the introductory letter

For the STOP CRC pilot, labels were printed in Excel using backend data, and clinic staff hand-stuffed letters. The clinic EMR site specialist used the “generate letters” function in Reporting Workbench to document that the letter was mailed to the selected list of patients. “Generate letters” date-stamped the mailing.

#### Step 2: Mailing FIT kits

To prepare the kits for mailing, the tool refined the original list of patients, filtering excluded patients who had an invalid address, those who called in to report prior screening, or those who declined participation. We assumed remaining patients had a valid address and were due for screening. Clinic staff used this list to (1) place laboratory orders for the tests; (2) print requisitions; (3) label the specimens; and (4) print labels and mailing the kits.

Placing the laboratory order for a patient eligible to receive the FIT kit was a relatively complex process. The Epic system allowed clinic staff to place a lab order without sending a specimen, using the “external interface, outside collection” order class. Working off the list of eligible patients, clinic staff placed laboratory orders for each patient, a critical function of the registry tool. Nevertheless, each order had to be placed one-at-a-time. Epic versions 2012 and later allow a user to place lab orders for a group of patients on a registry (batch ordering). This function will be available to OCHIN clinics in 2014 and a will be a useful part of the multi-site study. The EHR system automatically printed a requisition, which was included in the kit mailing so it would be returned to the laboratory with the collected sample. The EHR system also printed labels (containing patient name and health record numbers) to be affixed to the specimen collection tube. Once the specimen arrived at the lab and was processed, the lab result was routed via electronic interface to the ordering provider for review in the EHR, and evidence of the completed test was automatically populated into Reporting Workbench and Health Maintenance.

#### Step 3: Mailing a reminder postcard

The mailing of the reminder postcard was similar to the mailing of the letter. Briefly, “generate letters” was used to document the mailing for the subset of patients eligible for the reminder postcard. This subset included those who had not completed their kit with a valid address and who had not called in to report prior screening or to opt out. Staff created labels out of the EHR and mailed the cards.

#### Step 4: Delivering live telephone reminder calls

The research team developed a set of project-specific text macros in telephone notes (Smartphrases©) to record outcomes relevant for STOP CRC. Each macro was tied to a data element in the EHR for reporting and registry use. The macros included (1) needs new FIT kit; (2) opt out – declined; (3) opt out – previously screened; (4) opt out – other; and (5) clinical concerns. Clinic staff used the text macros to track incoming and outgoing phone calls. To record dispositions for outgoing reminder phone calls, we included an additional macro-- completed motivational interviewing phone call. Consistent with the standard clinic procedures, clinic staff attempt two telephone calls before considering the patient unreachable. The results of the telephone encounters were automatically populated into Reporting Workbench.

## Updating the registry and tracking CRC-relevant outcomes

As the automated intervention was a step-wise process, we updated the registry at each step to 1) eliminate patients who called in to opt out of the program or reported having been previously screened; 2) eliminate patients who had an invalid address that could not be corrected; and 3) track test completion and results, allowing the registry list to be filtered so that only those not completing screening received a reminder card and, in the case of Auto Plus, a motivational interviewing call.

The tool tracked the following CRC-relevant outcomes in the pilot phase of the study: completion of fecal testing, fecal test results, and completion of colonoscopy as a follow-up to an abnormal fecal test result. Chart audit validations revealed that 100% of lab data were correctly entered into the EHR. This facilitated real-time reporting and successful follow-up on patients with abnormal test results. The pilot clinics used existing workflows for tracking patient receipt of diagnostic colonoscopy.

## Training

On-site training was conducted by the EMR site specialist who demonstrated the tools and provided support to staff in using the tools. In addition, the purpose of the STOP CRC project was presented to the teams during 10 minutes of an on-going meeting. A bilingual staff motivational interviewer trained the patient care coordinator in using motivational interviewing techniques for the outgoing phone calls in the Auto Plus clinic. This training lasted one hour. The EMR specialist provided on-going support for questions and issues as they arose.

## Data collection

Our work sessions brought together project investigators and staff from all collaborating organizations (VGMHC, CHR-NW, Group Health Research Institute, and OCHIN). We held 3 work sessions, each lasting 4 hours. This was followed by hour-long meetings of the same group held every 1–2 weeks over a 6 month period. During these work sessions, we reviewed each step in the intervention, and identified what was done in the pilot, as well as what would change for the main study. In addition, our qualitative team conducted 9 in-person interviews with clinic staff who were involved in the pilot. Each interview lasted about 45 minutes and was conducted either in-person or over the phone. The findings of these interviews were discussed with the project team.

## Results

An important concern for STOP CRC was obtaining information on prior colonoscopy, a procedure that is captured inconsistently in health records of primary-care practices and rarely in discrete, searchable fields. Most colonoscopies are not performed in primary-care clinics, and instead are performed in specialty clinics, ambulatory centers, or hospitals. Thus, complete documentation of colonoscopy in primary care is often lacking. Clinics captured prior colonoscopy receipt in variable ways, such as via scans (which were sometimes coded by procedure), through the problem list, or via medical or surgical history sections of the EHR. This variability and the absence of a discrete field to document colonoscopies presented challenges to excluding patients who were not yet due for screening.

The pilot clinics used another Epic-embedded tool, Health Maintenance, to track outside screening events, including CRC screening. Health Maintenance uses results from interfaces such as completed FOBTs, and data manually entered by providers, to track screening events. Apart from tracking completed preventive health exams, such programs have the added advantage of making it possible to postpone preventive services in cases where patients have significant health conditions, are on hospice, or otherwise would be a poor candidate for preventive screening. As such, Health Maintenance, if used to identify eligible patients, allows personalized clinical decision-making to be incorporated into the selection of patients. Prior to our pilot study, Health Maintenance had recently been customized for colorectal cancer and made available for clinic use. Thus, it was not used to select eligible patients, but was used to update screening events that occurred as a result of the intervention or that were reported by patients who had previously completed screening.

Our research team identified some early limitations in the use of Health Maintenance to capture data for STOP CRC. First, documentation of a completed colonoscopy often omits a pathology report, the findings of which can determine the interval for future screening. Thus, the primary-care provider may be unaware of a given patient’s follow-up care plan and not have the necessary clinical information to appropriately postpone screening. Second, Health Maintenance searched only a limited number of fields in the EHR. OCHIN has planned to make future improvements to Health Maintenance, however, that will allow for searching multiple EHR fields (such as locating a historical order for a colonoscopy and the date it occurred) and relevant diagnosis codes (e.g. colorectal cancer, ulcerative colitis, etc.).

Moreover, having a direct interface with the laboratory that processes the test was considered a system requirement and critical to the management of the registry. Without timely and accurate data on results from fecal tests, the registry could not be updated to discontinue sending a reminder to patients who had completed screening.

Findings from Work Sessions and Qualitative Interviews.

During the work sessions, we developed consensus on several changes needed to improve the Reporting Workbench (Table 1). These changes were proposed either to overcome a limitation identified during the pilot, or to improve the usability of the system for the main study in which FIT kits will be mailed out at regular intervals (e.g. monthly or quarterly). We were also aware of two important system improvements that are slated to take place in parallel with our main study: improvements to Health Maintenance, which means that it will capture a greater percentage of previously screened patients; and a new release of Epic, which will offer the ability to order labs for a group of patients at one time. Several other revisions are planned. For the larger pragmatic study, we plan to create an option to print patient names and addresses on the backs of the introductory letters and use window envelopes, obviating the need to print address labels.

Interviews with clinic personnel revealed additional system challenges. First, several staff noted a greater need for training on the use of the tools overall and particularly in recording incoming and outgoing telephone calls. The text macros were considered not intuitive and were often incorrectly used in cases where patients returned a phone call initiated by clinic staff. Second, sending an additional FIT kit to patients who requested one was problematic, as the laboratory interface made it difficult to place a new lab order. Clinic staff also noted that several activities required more time than anticipated; this included setting up lab agreements with a new lab for processing the kits and placing the lab orders (as this was done one-by-one). The clinic also experienced issues with the laboratory interface which resulted in some patients being incorrectly billed for the test.

## Discussion

We found that most elements of our EHR-embedded program worked well and that specific refinement may improve the accuracy of identifying patients at each step. Our findings can inform future efforts to build EHR-embedded population management tools for preventive services.

Embedding the registry in the EHR offers multiple advantages over a stand-alone registry. An embedded registry allows changes in the type of test or diagnostic codes to be incorporated into the registry automatically (as they are incorporated into Health Maintenance). Another advantage is that staff only has to manage a single system. Even for an embedded system, however, comprehensive training and quality assurance is still required, to quality check routine reports of codes used and statuses obtained (e.g. prior colonoscopy screening).

The Health Maintenance tool will be improved in late 2013 based, in part, on observations from the STOP CRC pilot; specifically, the tool will be able to recognize historical CRC screening FIT and FOBT tests, and then update the registry report if a patient is not overdue for screening. Other improvements were made as a result of the findings from our pilot, developing multiple report templates rather than using a filter function; refining the text macros; and using a Best Practice Alert to integrate patient-reported information with Health Maintenance. Other upgrades will improve the efficiency of our program; the batch ordering of FIT tests requested by STOPC CRC sites will be possible when OCHIN upgrades to the newer version of the EHR in April 2014.

## Limitations

Challenges to the STOP CRC automated interventions include incomplete data-capture practices for manually entering data such as colonoscopies that occur outside of primary care. The documentation of colonoscopy varies by provider and can impact registry functionality. Standardizing processes for colonoscopy entry is problematic, even where there is direct data flow between primary care and specialty clinics and hospitals, as often much of this data is historical, predating a patient’s enrollment in a given clinic’s or health-care organization’s EHR implementation.

In the STOP study, we are working with our clinic partners and OCHIN to develop work flows to manage the collection of outside data. This will include standardizing methods for use of the Health Maintenance tool and for scanning of colonoscopies and similar documents. Collection of complete CRC data is not only critical for successful population-based screening, but also for good clinical care.

Apart from the requirements and key functions of the system, challenges to providing clinic-staff training to deliver the intervention and maintain the registry, remain. Clinic staff typically has limited non-clinical time set aside for training, and often the limited time they do have is dedicated to addressing clinical needs. Moreover, the need for point-of-care training, that is training that addresses questions as they occur, can be difficult to implement. On-going quality assurance is still needed to ensure that programming scripts and algorithms function as intended.

## Implications

There are several implications to having identified components of the tool to improve before beginning the larger multi-site trial we have planned. One is that it improves the chances that this evidence-based strategy can be easily adopted and maintained by health systems. A second implication is that it can improve the appropriate identification of patients who are eligible for each intervention step, and thereby maximize the efficient use of staff time and resources.

## Future directions

Our tool will be further tested in a large pragmatic study involving at least 24 FQHC clinics, set to begin in January 2014. Additional research is warranted to further maximize the ease of the use of the tools to support continued adoption.

## Figures and Tables

**Table 1:** Existing and planned EMR functionality for STOP CRC.

	EHR functionality used	EHR functionality planned
**Create a Patient Registry**	STOP CRC inclusion and exclusion codes were used (backend codes identified patients who had a clinic visit in past year, were aged 50–74, and were seen by selected PCPs, and were due for screening, and lacked significant co-morbid conditions).	Health Maintenance and STOP CRC inclusion and exclusion codes will be used; Health Maintenance will allow capture of patient diagnoses codes, and CRC screening data. This allows the registry to incorporate clinical decision-making as Health Maintenance can be manually postponed for patients who are poor candidates for screening.
**Step 1: Mail the Introductory Letter**	Documented mailing for a group of patients (“generate letters”); Printed labels.	Mail directly from the EHR for a group of patients.
**Updating the registry**	Use filtering in Reporting Workbench.	Use report templates for each step in the intervention that identifies patients eligible for that step.
**Step 2: Mail a FIT kit**	Placed lab orders for each patient individually; Printed labels mailing labels at OCHIN, using back-end data; Included requisition and bilingual instructions.	Place lab orders for a group of patients (batch orders); Print mailing labels from EHR or use windowed envelopes; Include requisition and wordless kit instructions.
**Step 3: Mail a Reminder Postcard**	Documented mailing for a group of patients; printed labels.	Mail directly from the EHR.
**Step 4: Deliver live phone calls**	Smart phrases to record phone outcomes.	Best Practice Alert to record phone outcomes.
